# Investigating the nexus of metabolic syndrome, serum uric acid, and dementia risk: a prospective cohort study

**DOI:** 10.1186/s12916-024-03302-5

**Published:** 2024-03-13

**Authors:** Tara SR Chen, Ning-Ning Mi, Hubert Yuenhei Lao, Chen-Yu Wang, Wai Leung Ambrose Lo, Yu-Rong Mao, Yan Tang, Zhong Pei, Jin-Qiu Yuan, Dong-Feng Huang

**Affiliations:** 1 Department of Rehabilitation Medicine, Guangdong Engineering and Technology Research Centre for Rehabilitation Medicine and Translation, The Seventh Affiliated Hospital, Sun Yat-Sen University, WHO Collaborating Centre for Rehabilitation CHN-50, Shenzhen, Guangdong China; 2 Department of Neurology, Guangdong Provincial Key Laboratory of Diagnosis and Treatment of Major Neurological Diseases, National Key Clinical Department and Key Discipline of Neurology, The First Affiliated Hospital, Sun Yat-Sen University, Guangzhou, Guangdong 510080 China; 3 Department of Epidemiology and Biostatistics, Clinical Big Data Research Centre, The Seventh Affiliated Hospital, Sun Yat-Sen University, Shenzhen, Guangdong China; 4 State Key Laboratory of Ophthalmology, Guangdong Provincial Key Laboratory of Ophthalmology and Visual Science, WHO Collaborating Centre for Eye Care and Vision CHN-151, Zhongshan Ophthalmic Center, Sun Yat-sen University, Guangzhou, 510060 China; 5 The First School of Clinical Medicine, Lanzhou University, Lanzhou, Gansu China; 6 Brain and Mind Centre, The University of Sydney, Sydney, Australia; 7 Department of Rehabilitation Medicine, The First Affiliated Hospital, Sun Yat-Sen University, Guangzhou, China

**Keywords:** Metabolic syndrome, Dementia, Alzheimer’s dementia, Vascular dementia, Risk factors, Serum uric acid

## Abstract

**Background:**

The global dementia prevalence is surging, necessitating research into contributing factors. We aimed to investigate the association between metabolic syndrome (MetS), its components, serum uric acid (SUA) levels, and dementia risk.

**Methods:**

Our prospective study comprised 466,788 participants without pre-existing MetS from the UK Biobank. We confirmed dementia diagnoses based on the ICD-10 criteria (F00-03). To evaluate the dementia risk concerning MetS, its components, and SUA levels, we applied Cox proportional hazards models, while adjusting for demographic factors.

**Results:**

Over a median follow-up of 12.7 years, we identified 6845 dementia cases. Individuals with MetS had a 25% higher risk of all-cause dementia (hazard ratio [HR] = 1.25, 95% confidence interval [CI] = 1.19–1.31). The risk increased with the number of MetS components including central obesity, dyslipidemia for high-density lipoprotein (HDL) cholesterol, hypertension, hyperglycemia, and dyslipidemia for triglycerides. Particularly for those with all five components (HR = 1.76, 95% CI = 1.51–2.04). Dyslipidemia for HDL cholesterol, hypertension, hyperglycemia, and dyslipidemia for triglycerides were independently associated with elevated dementia risk (*p* < 0.01). MetS was further linked to an increased risk of all-cause dementia (11%) and vascular dementia (VD, 50%) among individuals with SUA levels exceeding 400 μmol/L (all-cause dementia: HR = 1.11, 95% CI = 1.02–1.21; VD: HR = 1.50, 95% CI = 1.28–1.77).

**Conclusions:**

Our study provides robust evidence supporting the association between MetS, its components, and dementia risk. These findings emphasize the importance of considering MetS and SUA levels in assessing dementia risk, offering valuable insights for prevention and management strategies.

**Supplementary Information:**

The online version contains supplementary material available at 10.1186/s12916-024-03302-5.

## Background

Dementia, marked by progressive cognitive decline and functional impairment, poses a substantial public health challenge with limited curative options. The increasing global prevalence of dementia, projected to exceed 78 million by 2030 and potentially reach 139 million by 2050, underscores the crucial need to precisely elucidate its risk factors. Obesity, hypertension, and diabetes are recognized as modifiable risk factors for the onset and progression of dementia, as well as being core conditions associated with metabolic syndrome (MetS) [1].

MetS, characterized by dysglycemia, elevated blood pressure and triglyceride levels; low high-density lipoprotein cholesterol, and central obesity, is established as a risk factor for cardiovascular disease, coronary heart disease, and type 2 diabetes mellitus [2, 3]. Despite this recognition, the association between MetS and dementia risk exhibits variability across studies. In a mid-life cohort study, an incremental dementia risk was identified with each additional MetS component, albeit constrained by data limitations [4]. Similarly, a systematic review linked high waist circumference to cognitive impairment but lacked subtype-specific data [5]. Population-based studies and additional research strengthened MetS’ connection to Alzheimer’s disease (AD) [6, 7]. A nationwide exploration revealed a gradual increase in dementia risk with cumulative MetS components, while a cohort study associated MetS with incident vascular dementia (VD) [8, 9]. Persistent or worsening MetS components in a decade-long cohort study were linked to heightened dementia risk, although subtype assessments were lacking [10]. A recent investigation [11] disclosed a 12% increased risk of all-cause dementia with MetS, grappling with subgroup classifications. Conversely, a recent meta-analysis with nine longitudinal studies and 18,313 participants found no statistically significant association between MetS and incident dementia or AD [12]. Notably, the meta-analysis highlighted an increased incidence of pure VD and progression from mild cognitive impairment to dementia. In addition, elevated serum uric acid (SUA) consistently predicts the development of conditions like MetS, diabetes, hypertension, renal disease, cardiovascular disease (CVD), and CVD-related mortality in numerous prospective studies [13–19]. Studies on the relationship between MetS and dementia have indicated the potential influence of SUA [20, 21]. However, the findings are inconclusive and conflicting. SUA is under scrutiny due to its dual role as an antioxidant and potential pro-oxidant, prompting discussions about its connection to dementia risk with studies even suggesting that elevated SUA levels might slow AD progression [22–24]. A cohort study [25] revealed a correlation between high mid-life SUA and fast cognitive decline rather than risk of dementia, while hyperuricemia reduced the risk of vascular-type dementia [26]. Interestingly, gout patients exhibited a lower dementia risk [27], and a meta-analysis suggested AD risk with low UA concentrations [28]. In a cohort of 4618 participants aged 55 and older, higher SUA levels was found to be associated with reduced dementia risk [29]. Previous findings underscore limitations in understanding dementia subtypes and generalizability from small populations. Bridging these limitations is critical for a comprehensive grasp of how metabolic factors, including SUA, intersect with dementia risk, underscoring the need to explore specific dementia subtypes associated with MetS. To address these limitations, our study evaluates the association between MetS and its components in relation to the risk of incident dementia and its subtypes (AD and VD) in a population-based cohort of 466,377 participants observed over a median follow-up of 12.7 years. Additionally, our research explores the interaction between MetS and SUA in relation to the risks stratified by dementia subtypes.

## Methods

### Participants

**Fig. 1:**
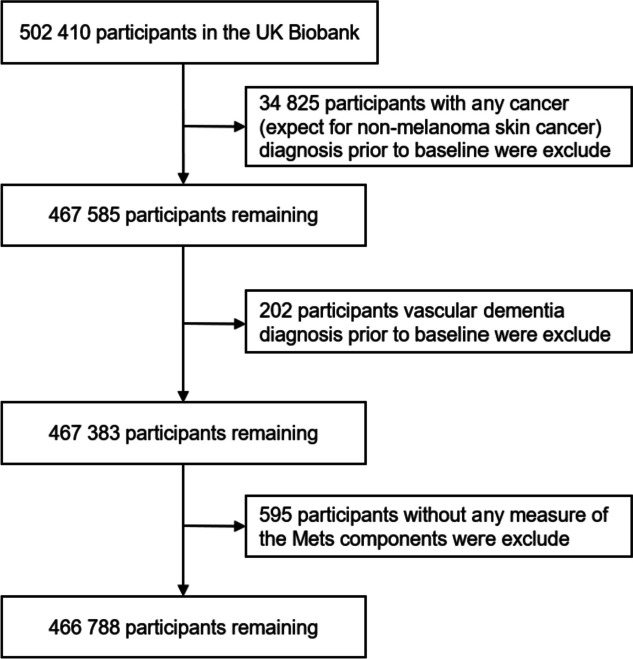
Participant inclusion flow diagram

Our study drew upon the UK Biobank's extensive dataset (https://www.ukbiobank.ac.uk), encompassing biological and medical information from approximately 500,000 adults aged between 40 and 70 years. Information regarding the study design and survey methods employed in the UK Biobank cohort can be found in previously published materials [30]. Ethical approvals were granted by the UK Biobank review committees (application number 51671, approved in August 2019). At baseline, 502,410 participants were recruited in this analysis. Participants with baseline cancer diagnoses (excluding non-melanoma skin cancer ICD-10 C44, *n* = 34,825) and pre-existing dementia diagnoses (*n* = 202) were excluded. Additionally, individuals with incomplete measurements for the five components of MetS (*n* = 595) were also excluded, resulting in a final analysis cohort of 466,788 participants, with a median follow-up of 12.7 years (Fig. 1).

### Assessment of outcomes

The diagnosis of dementia was determined through medical history and linkage to the hospital statistics from England, Scotland and Wales, utilizing the International Classification of Diseases (ICD-10), specifically F00-03. Participants eligible for the study contributed person-years from their date of enrolment until the first dementia diagnosis, date of death, or the last date of follow-up (December 31, 2021), whichever came first.

### Assessment of covariates

Participants completed a touchscreen questionnaire at the assessment center collect information on socio-demographic characteristics (i.e., age, gender, ethics, index of multiple deprivation), lifestyle factors (such as smoking status, alcohol consumption, physical activity, diet habits), and medication. Physical activity was assessed using adapted questions from the validated International Physical Activity Questionnaire (IPAQ), while dietary intake was assessed using a food frequency questionnaire, both of which have been validated in previous studies. During the physical examination, components of MetS were measured, including height, weight, waist circumference, and blood pressure. Fasting blood samples from each participant were collected by trained phlebotomists, and serum concentrations of glucose, high-density lipoprotein cholesterol, and triglycerides were measured using the Beckman Coulter AU5800 analyzer (Beckman Coulter (UK) Ltd., High Wycombe, UK). SUA was also measured using the Uricase PAP enzymatic method on the Beckman Coulter AU5800.

### Definition for metabolic syndrome (MetS)

The definition of MetS follows the International Diabetes Federation (IDF) criteria [31]. Participants with more than three components were classified as a MetS at baseline. According to the criteria, central obesity was defined as a waist circumference ≥ 94 cm for males or ≥ 80 cm for females. High blood pressure was defined as systolic blood pressure ≥ 130 mmHg or diastolic blood pressure ≥85 mmHg, or a prior diagnosis of hypertension under antihypertensive treatment. Elevated triglycerides were defined as plasma triglyceride (TG) levels ≥ 1.70 mmol/L (150 mg/dL) or currently receiving medication for hypertriglyceridemia. Low high-density lipoprotein (HDL) cholesterol was defined as male HDL < 1.03 mmol/L, female HDL < 1.29 mmol/L, or undergoing medication for lowering HDL cholesterol levels. High blood sugar was defined as fasting blood glucose ≥ 5.56 mmol/L or a previous diagnosis of type 2 diabetes.

### Statistical analysis

In descriptive analysis, numerical values were presented as the mean (standard deviation) for continuous variables or as numbers (percentages) for categorical variables. For covariates where respondents select “no response” or “don’t know,” or in cases of missing data, an “unknown/missing” response category was generated.

Cox proportional hazard regression analyses were performed to calculate the hazard ratio (HR) and 95% confidence interval (95% CI). The proportional hazards assumption was validated through Schoenfeld residuals. In basic model 1, we stratified jointly by age, gender, and UK Biobank assessment centers to estimate the crude associations of MetS and its components with dementia risk and its subtypes. We additionally adjusted for race (white or other), index of multiple deprivation (a measure of socioeconomic status), smoking status (never smoked, previous smoker, current smoker), alcohol consumption (never or special occasions only, one to three times a month, one to four times a week, daily or almost daily), physical activity (high, low, moderate or unknown/missing), portions of fruit and vegetable intake (< 5 portions per day, ≥ 5 portions per day, or unknown/missing), fish intake (< 3 times per week, ≥ 3 times per week) in model 2. In the fully adjusted model 3, we additionally adjusted for regular medications [multivitamin use (yes or no), mineral supplement use (yes or no), non-steroidal anti-inflammatory drugs use (yes or no), aspirin use (yes or no)], and history of Alzheimer’s disease/dementia (yes or no). Additionally, the restricted cubic method was employed to evaluate the potential non-linearity association of each MetS component with the risk of dementia. We used a restricted cubic spline regression model with three knots at the 10th, 50th, and 90th percentiles of each MetS component to achieve the best fit. Non-linearity associations were investigated by using a likelihood ratio test comparing the model with only a linear term against the model with linear and cubic spline terms. To assess the joint effect of SUA on the association between MetS and dementia subtypes, four risk levels based on MetS (presence or absence) or SUA (with an SUA cut-off at 400 μmol/L) [32] and HRs for dementia subtypes risk were calculated with reference to “No MetS and SUA < 400 μmol/L group”. To further investigate potential effect modifiers, we conducted subgroup analyses stratified by age, sex, smoking habits, alcohol consumption, physical activity, fruit and vegetable intake, regular use of aspirin, and non-steroidal anti-inflammatory. In sensitivity analysis, we lagged the exposure for 2 years to avoid potential reverse causation. All statistical tests were two-tailed, and significance was defined as *p* < 0.05. Statistical analyses were conducted using the R software (version 4.1.0, R Foundation for Statistical Computing, Vienna, Austria).

## Results

In our analysis encompassing 466,788 participants (46.4% male, 53.6% female, Table 1), we observed a higher prevalence of former smokers and lower physical activity and fruit/vegetable intake among the MetS cohort. Participants with MetS tended to be older and have a higher multiple deprivation index, higher BMI, blood pressure and serum concentrations of triglyceride, fasting glucose, and lower serum concentrations of HDL-cholesterol than the non-MetS group.

**Table 1:** Baseline characteristics of participants stratified by metabolic syndrome status in the UK Biobank cohort

**Characteristics**	**Non-metabolic syndrome** **(*N* = 354 625)**	**Metabolic syndrome** **(*N* = 112 163)**	**Overall (*N* = 466 788)**
Mean (SD) age, years	56.2 (8.2)	58.5 (7.7)	56.8 (8.1)
Female, *N* (%)	194,126 (54.7)	56,185 (50.1)	250,311 (53.6)
White, *N* (%)	334,812 (94.4)	106,066 (94.6)	440,878 (94.4)
Mean (SD), index of multiple deprivation	16.6 (13.6)	19.6 (15.3)	17.3 (14.1)
Never smoker	201,094 (56.7)	54,663 (48.7)	255,757 (54.8)
Never drinker	25,588 (7.2)	11,765 (10.5)	37,353 (8.0)
> 5 portions of fruit and vegetable per day, *N* (%)	135,273 (38.1)	40,167 (35.8)	175,440 (37.6)
High activity, *N* (%)	122,911 (34.7)	28,614 (25.5)	151,525 (32.5)
Multivitamin use, *N* (%)	53,834 (15.2)	15,467 (13.8)	69,301 (14.8)
Intake of mineral supplements, *N* (%)	77,332 (21.8)	21,972 (19.6)	99,304 (21.3)
Aspirin use, *N* (%)	38,800 (10.9)	27,687 (24.7)	66,487 (14.2)
Non-aspirin NSAIDs use, *N* (%)	56,495 (15.9)	19,592 (17.5)	76,087 (16.3)
History of Alzheimer’s disease/dementia	41,322 (11.7)	12,983 (11.6)	54,305 (11.6)
Mean (SD) waist circumference, cm	86.0 (11.1)	104 (10.8)	90.3 (13.5)
Mean (SD) BMI, kg/m^2^	25.9 (3.71)	32.4 (4.4)	27.4 (4.8)
Mean (SD) TG, mmol/L	1.52 (0.9)	2.40 (1.2)	1.74 (1.0)
Mean (SD) HDL, mmol/L	1.52 (0.4)	1.23 (0.3)	1.45 (0.4)
Mean (SD) LDL, mmol/L	3.56 (0.8)	3.53 (1.0)	3.55 (0.867)
Mean (SD) cholesterol, mmol/L	5.73 (1.1)	5.57 (1.3)	5.69 (1.1)
Mean (SD) fasting glucose, mmol/L	4.97 (0.9)	5.54 (1.8)	5.12 (1.2)
Mean (SD) HbA1c, mmol/L	35.1 (5.2)	39.3 (9.6)	36.1 (6.8)
Mean (SD) SBP, mmHg	138 (19.7)	146 (18.0)	140 (19.7)
Mean (SD) DBP, mmHg	81.1 (10.6)	86.0 (10.3)	82.3 (10.7)

*Abbreviations*: *BMI* body mass index, *TG* triglyceride, *HDL* high-density lipoprotein, *LDL* low-density lipoprotein, *SBP* systolic blood pressure, *DBP* diastolic blood pressure, *HbA1c* hemoglobin A1c, *NASIDS* non-steroidal anti-inflammatory drugs, *ASP* aspirin

**Fig. 2:**
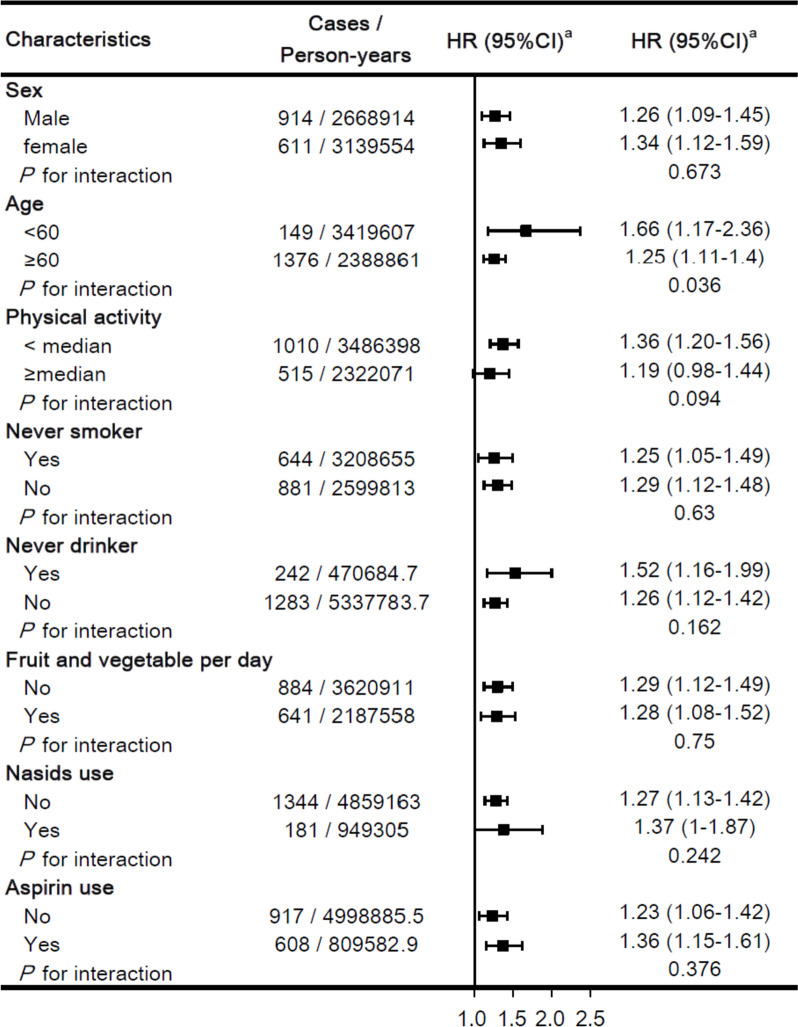
Subgroup analyses of metabolic syndrome components and dementia risk. ^a^ Subgroup analyses stratified by age, gender, and UK Biobank assessment centers. Adjusted for various factors, including race, deprivation index, lifestyle habits, and medication history

During a median follow-up period of 12.7 years, we observed 6,845 cases of dementia. Table 2 presents the significant association between MetS and dementia, including its subtypes. In general, individuals with MetS exerted a 25% higher risk of all-cause dementia (HR = 1.25, 95% CI = 1.19–1.31), a trend consistent across AD and VD. However, after comprehensive adjustments (sociodemographic characteristics, lifestyle factors, medications, and dementia history), the association with AD weakened (HR = 0.98, 95% CI = 0.90–1.07), while all-cause dementia (HR = 1.06, 95% CI = 1.01–1.12) and VD (HR=1.28, 95% CI = 1.15–1.43) associations remained significant. Subgroup analyses yielded consistent risk estimates, demonstrating the robustness of the association between MetS and all-cause dementia across various risk factors, including age, sex, smoking and drinking status, physical activity, fruit and vegetable intake, NSAIDS use, and aspirin use (Fig. 2). In a sensitivity analysis incorporating a 2-year exposure lag, the positive association persisted between MetS and all-cause dementia as well as VD (Additional file 1: Table S1).

**Table 2:** Association between metabolic syndrome and dementia risk

**Variable**	**All-cause dementia**		**Alzheimer’s disease**		**Vascular dementia**	
	**Non-MetS**	**MetS**	**Non-MetS**	**MetS**^**e**^	**Non-MetS**	**MetS**^**e**^
Cases, *n*	4597	2248	2002	883	925	600
Person-years	4,422,910	1,374,613	4,428,013	1,377,299	4,430,434	1,378,035
Incidence rate^a^	103.94	163.54	45.21	64.11	20.88	43.54
Model 1^b^, HR (95% CI)	1.00 (Ref)	1.25 (1.19–1.31)*	1.00 (Ref)	1.11 (1.02–1.20)*	1.00 (Ref)	1.64 (1.48–1.82)*
Model 2^c^, HR (95% CI)	1.00 (Ref)	1.11 (1.05–1.17)*	1.00 (Ref)	1.01 (0.93–1.09)	1.00 (Ref)	1.41 (1.27–1.57)*
Model 3^d^, HR (95% CI)	1.00 (Ref)	1.06 (1.01–1.12)*	1.00 (Ref)	0.98 (0.90–1.07)	1.00 (Ref)	1.28 (1.15–1.43)*

*MetS*, metabolic syndrome

^*^P-value < 0.05

^a^Per 100,000 person-years

^b^Model 1, stratified by age, gender, and UK Biobank assessment center

^c^Model 2, additionally adjusted for race (white or other), index of multiple deprivation (a measure of socioeconomic status), smoking status (never smoked, previous smoker, current smoker), alcohol consumption (never or special occasions only, one to three times a month, one to four times a week, daily or almost daily), physical activity (high, low, moderate, or unknown/missing), portions of fruit and vegetable intake (< 5 portions per day, ≥ 5 portions per day, or unknown/missing)

^d^Model 3, fully adjusted model additionally adjusted for regular medications [multivitamin use (yes or no), mineral supplement (yes or no), non-steroidal anti-inflammatory drugs (yes or no), aspirin (yes or no)], and history of Alzheimer’s disease/dementia (yes or no)

The risk of dementia increased with an increasing number of MetS components (*p* trend < 0.001). Participants manifesting all five abnormal components experienced significantly elevated risks of all-cause dementia (HR = 1.76, 95% CI = 1.51–2.04) and VD (HR = 3.72, 95% CI = 2.64–5.24). Among the individual MetS components, dyslipidemia for HDL cholesterol (HR = 1.07, 95% CI = 1.01–1.14), hypertension (HR = 1.09, 95% CI = 1.02–1.17), hyperglycemia (HR = 1.43, 95% CI = 1.35–1.51), and dyslipidemia for triglycerides (HR = 1.05, 95% CI = 1.00–1.11) exhibited significant positive associations with dementia risk (*p* < 0.01). Moreover, all MetS components were associated with a higher risk of VD (Table 3).

**Table 3:** Risk of dementia according to each component of metabolic syndrome

**Variable**	**All-cause dementia**			**Alzheimer’s disease**			**Vascular dementia**		
	**Model 1**	**Model 2**	**Model 3**	**Model 1**	**Model 2**	**Model 3**	**Model 1**	**Model 2**	**Model 3**
**Central obesity**									
No	1.00 (Ref)	1.00 (Ref)	1.00 (Ref)	1.00 (Ref)	1.00 (Ref)	1.00 (Ref)	1.00 (Ref)	1.00 (Ref)	1.00 (Ref)
Yes	1.15 (1.10–1.21)*	1.03 (0.98–1.08)	1.00 (0.96–1.06)	1.00 (0.92–1.07)	0.91 (0.85–0.99)	0.90 (0.83–0.98)	1.44 (1.30–1.60)*	1.25 (1.13–1.39)*	1.18 (1.06–1.31)*
**Dyslipidemia for HDL cholesterol**									
No	1.00 (Ref)	1.00 (Ref)	1.00 (Ref)	1.00 (Ref)	1.00 (Ref)	1.00 (Ref)	1.00 (Ref)	1.00 (Ref)	1.00 (Ref)
Yes	1.29 (1.22–1.37)*	1.11 (1.04–1.18)*	1.07 (1.01–1.14)*	1.19 (1.08–1.30)*	1.04 (0.95–1.15)	1.02 (0.93–1.13)	1.62 (1.43–1.82)*	1.33 (1.17–1.51)*	1.24 (1.09–1.41)*
**Hypertension**									
No	1.00 (Ref)	1.00 Ref)	1.00 (Ref)	1.00 (Ref)	1.00 (Ref)	1.00 (Ref)	1.00 (Ref)	1.00 (Ref)	1.00 (Ref)
Yes	1.13 (1.05–1.21)*	1.12 (1.04–1.2)*	1.09 (1.02–1.17)*	1.16 (1.04–1.29)*	1.16 (1.03–1.29)*	1.15 (1.03–1.28)*	1.44 (1.22–1.70)*	1.42 (1.20–1.68)*	1.33 (1.12–1.58)*
**Hyperglycemia**									
No	1.00 (Ref)	1.00 (Ref)	1.00 (Ref)	1.00 (Ref)	1.00 (Ref)	1.00 (Ref)	1.00 (Ref)	1.00 (Ref)	1.00 (Ref)
Yes	1.59 (1.50–1.68)*	1.48 (1.40–1.57)*	1.4 (1.35–1.51)*	1.38 (1.26–1.50)*	1.30 (1.19–1.42)*	1.27 (1.16–1.39)*	2.26 (2.02–2.52)*	2.03 (1.81–2.27)*	1.85 (1.65–2.07)*
**Dyslipidemia for triglycerides**									
No	1.00 (Ref)	1.00 (Ref)	1.00 (Ref)	1.00 (Ref)	1.00 (Ref)	1.00 (Ref)	1.00 (Ref)	1.00 (Ref)	1.00 (Ref)
Yes	1.20 (1.14–1.27)*	1.12 (1.06–1.18)*	1.05 (1.00–1.11)*	1.16 (1.07–1.26)*	1.09 (1.01–1.19)*	1.06 (0.97–1.15)	1.65 (1.47–1.86)*	1.51 (1.34–1.70)*	1.33 (1.17–1.50)*
**Number of MetS components**									
0	1.00 (Ref)	1.00 (Ref)	1.00 (Ref)	1.00 (Ref)	1.00 (Ref)	1.00 (Ref)	1.00 (Ref)	1.00 (Ref)	1.00 (Ref)
1	1.19 (1.06–1.34)*	1.19 (1.06–1.34)*	1.19 (1.06–1.34)*	1.23 (1.02–1.46)*	1.23 (1.02–1.47)*	1.23 (1.03–1.47)*	1.50 (1.10–2.04)*	1.49 (1.09–2.04)*	1.48 (1.08–2.02)*
2	1.23 (1.09–1.38)*	1.19 (1.06–1.34)*	1.16 (1.03–1.30)*	1.19 (1.00–1.42)*	1.17 (0.98–1.41)	1.16 (0.97–1.39)	1.73 (1.27–2.34)*	1.66 (1.22–2.26)*	1.55 (1.14–2.11)*
3	1.40 (1.25–1.58)*	1.27 (1.13–1.43)*	1.20 (1.07–1.36)*	1.36 (1.13–1.62)*	1.26 (1.05–1.51)*	1.22 (1.01–1.47)*	2.22 (1.63–3.01)*	1.96 (1.43–2.67)*	1.73 (1.27–2.37)*
4	1.66 (1.46–1.88)*	1.42 (1.25–1.61)*	1.32 (1.16–1.50)*	1.43 (1.18–1.74)*	1.27 1.04–1.55)*	1.22 (1.00–1.49)*	3.02 (2.20–4.13)*	2.52 (1.83–3.46)*	2.14 (1.55–2.96)*
5	2.49 (2.15–2.88)*	1.95 (1.68–2.26)*	1.76 (1.51–2.04)*	1.97 (1.56–2.48)*	1.63 (1.28–2.06)*	1.53 (1.20–1.94)*	6.28 (4.51–8.74)*	4.67 (3.33–6.56)*	3.72 (2.64–5.24)*
	*p* trend <0.001	*p* trend <0.001	*p* trend <0.001	*p* trend <0.001	*p* trend =0.002	*p* trend =0.029	*p* trend <0.001	*p* trend <0.001	*p* trend <0.001

Model 1, stratified by age, gender, and UK Biobank assessment center

Model 2, additionally adjusted for race (white or other), index of multiple deprivation (a measure of socioeconomic status), smoking status (never smoked, previous smoker, current smoker), alcohol consumption (never or special occasions only, one to three times a month, one to four times a week, daily or almost daily), physical activity (high, low, moderate or unknown/missing), portions of fruit and vegetable intake (< 5 portions per day, ≥ 5 portions per day, or unknown/missing)

Model 3, fully adjusted model additionally adjusted for regular medications [multivitamin use (yes or no), mineral supplement (yes or no), non-steroidal anti-inflammatory drugs (yes or no), aspirin (yes or no)], and history of Alzheimer’s disease/dementia (yes or no)

^*^P-value < 0.05

Exploring the interaction between MetS and SUA levels, we found that MetS was linked to an elevated risk of all-cause dementia and VD in both groups stratified by SUA levels. Notably, the risk substantially escalated in participants with both MetS and SUA levels exceeding 400 μmol/L (all-cause dementia: HR = 1.11, 95% CI = 1.02–1.21; VD: HR = 1.50, 95% CI = 1.28–1.77) when compared to participants without MetS and SUA levels at or below 400 μmol/L in the fully adjusted model 3 (Table 4).

**Table 4:** Risk of dementia with the joint effect of serum uric acid and metabolic syndrome

	**All-cause dementia**			**Alzheimer’s disease**			**Vascular dementia**		
	**Model 1**	**Model 2**	**Model 3**	**Model 1**	**Model 2**	**Model 3**	**Model 1**	**Model 2**	**Model 3**
**Uric acid ≤ 400**									
Non-MetS	Ref	Ref	Ref	Ref	Ref	Ref	Ref	Ref	Ref
MetS	1.22 (1.15–1.29)*	1.07 (1.01–1.14)*	1.03 (0.97–1.10)	1.09 (1.00–1.20)	0.99 (0.90–1.08)	0.96 (0.88–1.06)	1.57 (1.39–1.77)*	1.34 (1.18–1.52)*	1.23 (1.08–1.40)*
**Uric acid > 400**									
Non-MetS	Ref	Ref	Ref	Ref	Ref	Ref	Ref	Ref	Ref
MetsS	1.35 (1.21–1.49)*	1.23 (1.11–1.37)*	1.17 (1.05–1.30)*	1.16 (0.98–1.37)	1.09 (0.92–1.30)	1.07 (0.89–1.27)	1.77 (1.45–2.16)*	1.56 (1.27–1.92)*	1.39 (1.13–1.71)*
**Joint effect of MetS and uric acid**									
Non-MetS SUA ≤ 400	Ref	Ref	Ref	Ref	Ref	Ref	Ref	Ref	Ref
Non-MetS SUA > 400	0.96 (0.89–1.04)	0.95 (0.87–1.03)	0.95 (0.87–1.02)	0.94 (0.83–1.06)	0.94 (0.83–1.06)	0.94 (0.83–1.06)	1.11 (0.94–1.31)	1.09 (0.92–1.28)	1.08 (0.91–1.27)
MetS SUA ≤ 400	1.22 (1.15–1.29)*	1.07 (1.01–1.14)*	1.03 (0.97–1.10)	1.09 (1.00–1.20)*	0.99 (0.90–1.08)	0.96 (0.88–1.06)	1.57 (1.39–1.77)*	1.34 (1.18–1.52)*	1.23 (1.08–1.40)*
MetS SUA > 400	1.29 (1.19–1.41)*	1.17 (1.07–1.27)*	1.11 (1.02–1.21)*	1.10 (0.96–1.26)	1.02 (0.88–1.17)	0.98 (0.85–1.13)	1.94 (1.66–2.28)*	1.69 (1.44–1.99)*	1.50 (1.28–1.77)*

^*^P-value < 0.05

Model 1, stratified by age, gender, and UK Biobank assessment center

Model 2, additionally adjusted for race (white or other), index of multiple deprivation (a measure of socioeconomic status), smoking status (never smoked, previous smoker, current smoker), alcohol consumption (never or special occasions only, one to three times a month, one to four times a week, daily or almost daily), physical activity (high, low, moderate or unknown/missing), portions of fruit and vegetable intake (< 5 portions per day, ≥ 5 portions per day, or unknown/missing) and fish intake (< 3 times per week, ≥ 3 times per week)

Model 3, fully adjusted model additionally adjusted for regular medications [multivitamin use (yes or no), mineral supplement (yes or no), non-steroidal anti-inflammatory drugs (yes or no), aspirin (yes or no)], and history of Alzheimer’s disease/dementia (yes or no)

**Fig. 3:**
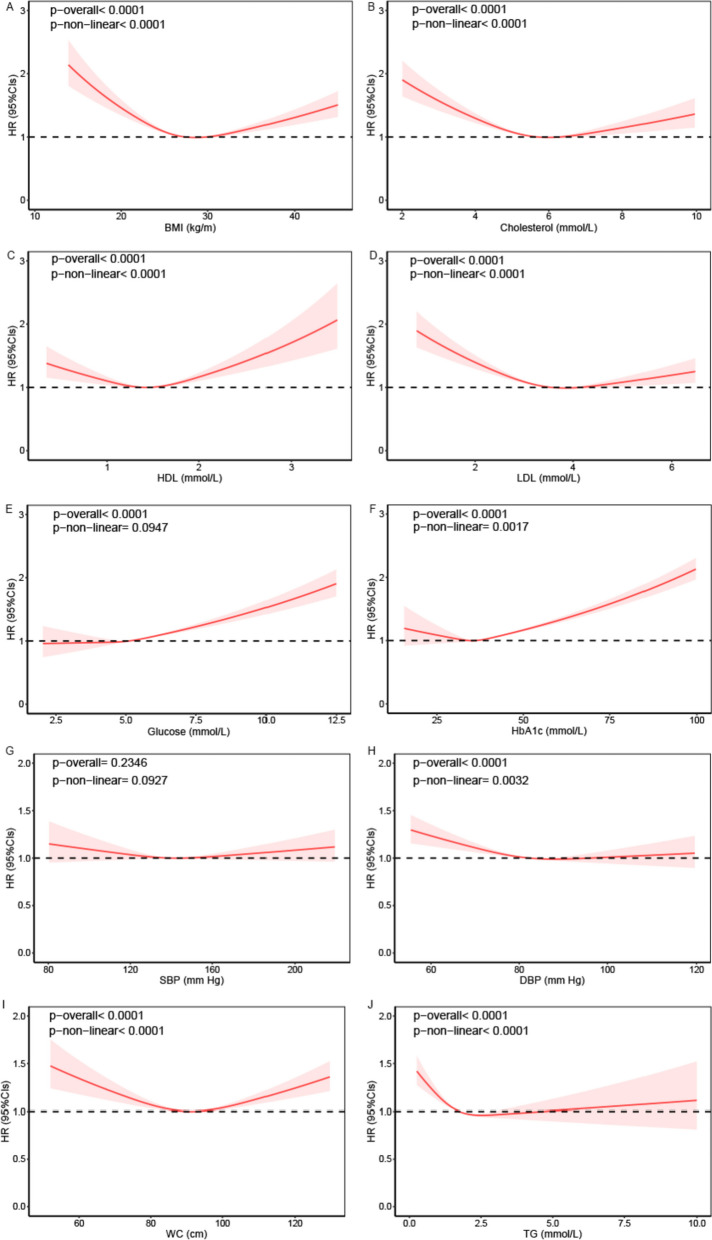
Non-linear association between metabolic syndrome components and all-cause dementia risk. Restricted cubic spline models, with knots at the 10th, 50th, and 90th percentiles, were employed. Reference levels (HR fixed at 1.0) for each plot: **A** BMI: 27.42 kg/m^2^; **B** cholesterol: 5.69 mmol/L; **C** HDL: 1.45 mmol/L; **D** LDL: 3.56 mmol/L; **E** glucose: 5.12 mmol/L; **F** HbA1c: 36.08 mmol/L; **G** SBP: 139.6 mm Hg; **H** DBP: 82.25 mm Hg; **I** WC: 90.27 cm; **J** TG: 1.74 mmol/L Adjustments were made for age, gender, and UK Biobank assessment centers. Additional adjustments included race, index of multiple deprivation, smoking status, alcohol consumption, physical activity, portions of fruit and vegetable intake, regular medications (multivitamin use, mineral supplement, non-steroidal anti-inflammatory drugs, and aspirin), and history of dementia

Furthermore, our analysis of the non-linear associations of continuous individual MetS components with dementia risk unveiled that higher fasting glucose was linked to significantly heightened risks of all-cause dementia, without evidence of non-linearity. Conversely, U-shaped patterns characterized the associations between other MetS components and dementia risk. Similar results were observed for the associations of MetS components with VD risk (Fig. 3 and Additional file 1 Figure S1).

## Discussion

In this comprehensive study utilizing UK Biobank data, we explored the association between MetS and dementia, focusing on VD and AD. Our analysis encompassed data from 466,788 adults aged 40 to 70 years with genders balanced. The study unveiled a substantial 25% increased risk of all-cause dementia associated with MetS. Notably, this association exhibited a more pronounced effect for VD than AD. The risks for AD became apparent only after a 2-year exposure lag. This lag was strategically incorporated into our study design to account for potential delays in the manifestation of dementia symptoms following exposure to MetS. Additionally, as individuals accumulated abnormal metabolic components, their risk of both VD and all-cause dementia increased. Elevated SUA levels above 400 μmol/L were associated with a higher risk of all-cause dementia and VD, but not AD. These findings underscore the significant relationship between MetS and SUA in dementia risk and highlight the importance of considering these factors across different dementia subtypes.

Our findings contribute to the body of evidence on the relationship between MetS components and dementia risk. Confirming prior research on the association between MetS and dementia risk [4, 33]. We affirm that individuals with MetS face a heightened risk of dementia [4, 10, 11], particularly in the case of VD [9, 12, 34, 35] and AD [6, 7]. This risk escalated progressively with each additional MetS component. Discrepancies arise in previous studies regarding the association between MetS and AD [7, 9]. In a comprehensive four-year study conducted in France involving 7,078 subjects found no substantial association between MetS and AD [9]. Conversely, an alternative investigation yielded dissimilar results, disclosing a positive correlation between MetS and AD while failing to establish a significant link with VD [7]. This divergence in results can be ascribed, at least partially, to disparities in demographic composition, study design, methodology, and sample size. Such that, the study exclusively focused on a cohort of 84,144 participants with an average age of 67 years, a population segment renowned for a notable increase in AD incidence with advancing age [36, 37]. Our study encompasses a larger cohort with a balanced gender distribution and an average age of 56.8 years which is more representative of the age range at which dementia risk assessments are typically conducted. We also took into account the multifactorial nature of MetS, subject to influences from lifestyle and mental health status [38–40], our analysis strikes a methodological balance, capturing diverse participants while mitigating susceptibility to reverse causation bias over an extended follow-up period of 12.7 years.

Examining modifiable factors associated with MetS, we uncovered a higher prevalence of former smokers and lower physical activity and fruit/vegetable intake among the MetS cohort. These associations imply potential modifications in cognitive reserve with the addition of each MetS component. Furthermore, we accounted for medication use, adjusting for NSAIDs [41], a critical factor as it may have influenced the observed reduction in dementia cases. Our analysis implies individuals with MetS, particularly those exhibiting four or five components, may derive potential benefits from early intervention and prevention measures to mitigate the risk of dementia.

Moving beyond the broad link between MetS and dementia, our analysis unveils a nuanced, non-linear relationship between individual MetS components and dementia risk. While higher fasting glucose and systolic blood pressure show linear associations, others, exhibit U-shaped patterns. This dynamic, possibly due to a threshold effect, implies a significant increase in dementia risk beyond specific MetS levels. The interplay of each component contributes significantly to this non-linear nature. Moreover, individual variability, influenced by lifestyle factors such as diet, physical activity, and waist circumference, may play a substantial role. Understanding this relationship demands comprehensive consideration of multifaceted factors, emphasizing the necessity for sophisticated approaches in both research and clinical practice. This underscores the notion that the connections between MetS components and dementia risk surpass a simplistic linear correlation.

In our analysis with a particular focus on VD concerning dyslipidemia for HDL cholesterol, hyperglycemia, and dyslipidemia for triglycerides, along with hypertension, it is evident that each of these factors independently contributes to an elevated risk of VD. These findings underscore the substantial impact of metabolic factors on the risk of dementia, even when considering other components of MetS. Regarding AD, we initially observed an association in the unadjusted model (HR = 1.11 95% CI: 1.02–1.20). However, this association lost statistical significance after accounting for various factors, including anti-inflammatory drug usage. This implies a potential role for anti-inflammatory drugs in mitigating AD risk and its associated symptoms within the dementia spectrum, warranting further investigation.

Refining our analysis of dementia subtypes, atherogenic dyslipidemia emerges as a noteworthy factor in dementia risk. The substantial association with dementia, particularly VD (HR = 1.33 (95% CI: 1.17–1.50), underscores its impact on the overall risk profile. This vascular dysfunction contributes to atherosclerotic lesions, particularly in carotid and vertebrobasilar systems, causing chronic hemispheric hypoperfusion [42, 43]. The heightened dementia risk persists even in cases of minor strategic infarcts within vital cognitive regions, as identified through voxel-based brain MRI analysis [44, 45].

Furthermore, hypertension emerges as another critical factor, with HRs of 1.09 (95% CI 1.02–1.17) for all-cause dementia, 1.15 (95% CI 1.03–1.28) for AD, and 1.33 (95% CI 1.12–1.58) for VD. The pronounced dementia risk associated with hypertension, particularly for VD, underscores its role in inducing early cerebral blood flow dysfunction and vascular remodeling [42, 46–50]. Cerebral small vessel disease, often prevalent in individuals with hypertension, predominantly affects white matter integrity due to various factors, including endothelial dysfunction, hypoperfusion, and blood-brain barrier disruption, leading to cerebral atrophies, and eventually cognitive dysfunction [42]. Our results also indicated that each MetS component is independently associated with an increased risk of AD, with a significance level of *p*-value < 0.05 over the five accumulative effects of the MetS components. Our study reaffirms the potential pathogenic interrelationships between metabolic factors especially hypertension to cognitive health.

Considering inflammation as a potential common risk factor in both MetS and dementia, recent studies proposed a pivotal role for SUA in atherosclerosis [51] and its involvement in triggering systemic inflammation in MetS [52]. Despite the controversial dual role of SUA, our findings align with previous research indicating that elevated SUA levels, not in the context of MetS, are associated with an increased risk of VD [53]. Our analysis underscores a correlation between elevated SUA levels (> 400 μmol/L) and a heightened risk of all-cause dementia with HRs of 1.11 (95% CI 1.02–1.21), and VD with an increased HR of 1.50 (95% CI 1.28–1.77). This emphasizes the significant impact of elevated SUA on VD risk. Indeed, SUA’s role in atherosclerosis and its link to hippocampal inflammation suggests a potential avenue for future research into neuroinflammation, given the established role of the hippocampus in dementia development. Unlike previous studies solely investigating the connection between dementia risk across scaled urate ranges [54], our research comprehensively considers all five MetS components to dementia subtypes. Recognizing the limitations of using a single index [54], such as BMI, for assessing the impact of MetS, especially beyond three components, highlights the novel approach our study undertakes. Additionally, our analysis’ equal gender distribution minimizes potential gender bias. Suggesting interventions for dementia, considering gender and serum uric acid variations, necessitates personalized strategies to accommodate diverse responses and specific factors influencing treatment efficacy [55–61]. Furthermore hyperuricemia, characterized by elevated serum uric acid, is associated with well-established risk factors for dementia including cardiovascular disease, diabetes, and hypertension—and links to dementia [62]. While our study did not find an increased risk of AD associated with elevated SUA levels, the potential antioxidant neuroprotective role of SUA [63] is implicated in our results, suggesting SUA could be a potential biomarker for monitoring dementia development among MetS individuals—a novel avenue for future investigation.

Our research unravels the intricate relationship between SUA and dementia risk, particularly in the realms of AD and VD. Elevated SUA levels (> 400 μmol/L) reveal a robust and significant association, indicating an 11% heightened risk of all-cause dementia and a substantial 50% increased risk of VD among individuals with MetS. Interestingly, no significant association is observed with AD, suggesting a potential antioxidative effect of higher SUA levels on cognitive health possibly contributing to an overall reduction in AD risk. These findings align with previous research on SUA’s association with its potential neuroprotective role [64, 65]. Nevertheless, it is important to note that the relationship between SUA and AD remains a subject of controversy [21]. Our study enriches the growing body of evidence concerning the interplay between MetS, its components, and dementia risk. The identification of non-linear associations observed for specific MetS components, along with the varied impacts on different dementia subtypes, underscores the importance of personalized approaches in dementia risk assessment and management. These insights are invaluable for developing comprehensive care strategies that address both metabolic health and dementia prevention, aligning with global health initiatives focused on dementia care and prevention. Our findings align with global health initiatives focused on enhancing dementia care and prevention, underscoring the importance of deciphering these complex relationships for the improvement of public health.

To the best of our knowledge, our study is the first to comprehensively explore the association between SUA, MetS, and the risk of AD, VD, and all-cause dementia subtypes. This research introduces novelty and significance to the field, and ongoing scientific exploration and dialog are crucial for refining our understanding of these intricate relationships and their implications for public health.

## Strength and limitations

In our study using UK Biobank data to assess the relationship between MetS and dementia, we recognize several limitations. The participant cohort, mainly from the UK, may not represent global populations, impacting the generalizability of our findings. This is especially relevant given the varied influences of genetic, gender, lifestyle, nutrition status, and environmental factors on MetS and dementia risks across different demographics. Being observational, our study establishes associations but not causality, and there is a potential for unmeasured confounding factors. The use of baseline measurements for SUA and MetS components might not reflect changes over the 12.7-year follow-up period. This is significant for diseases like Alzheimer’s, which develop progressively. While reverse causality is less likely, it's not entirely ruled out. Data reliability could be influenced by self-reported information and changes in diagnostic criteria. Participant dropouts or loss to follow-up may also bias the results. Furthermore, our findings may not apply to diverse age groups, ethnicities, or healthcare systems. Some subgroup analyses might have limited statistical power, affecting the robustness of conclusions.

These limitations emphasize the need for cautious interpretation of our results and suggest the value of future research with more diverse populations and dynamic measurements for a deeper understanding of the MetS-dementia relationship.

## Conclusions

In conclusion, our extensive analysis of 466,788 UK Biobank participants reveals a nuanced relationship between MetS and dementia. We observe a 25% increased risk of all-cause dementia, with a more pronounced association for VD over AD. The emergence of AD risk becomes apparent after a 2-year exposure lag providing valuable insights into temporal dynamics. As participants accumulate abnormal metabolic components, risks for VD and all-cause dementia rise, particularly with elevated SUA levels above 400 μmol/L, highlighting the significant association between MetS, SUA, and dementia risk across subtypes. Notably, elevated SUA levels are associated with an 11% increased risk of all-cause dementia and a 50% increased risk of VD. Our study contributes novel insights by examining the cumulative impact of all five MetS components and SUA levels on VD risk. The identification of non-linear associations for specific MetS components underscores the necessity for personalized approaches in dementia risk assessment. Comprehensively examining a large cohort, our study distinguishes itself by moving beyond singular components and addressing potential delays in symptom manifestation, enhancing the accuracy of assessing the temporal relationship between MetS and AD.

In summary, our study reaffirms the MetS-dementia association while offering novel insights. The diverse impacts on different dementia subtypes, the influence of SUA, and the strategic lag contribute to a comprehensive understanding. Advocating for a shift toward personalized, targeted approaches in dementia risk evaluation and intervention, our findings align with global health initiatives for dementia care and prevention.

### Supplementary Information

**Additional file 1:** **Table S1.** The association between metabolic syndrome and risk of dementia after lagging for 2 years. **Figure S1.** Non-linear Association Between Metabolic Syndrome Components and Vascular Dementia Risk.

## Data Availability

The data that support the findings of this study are available from the UK Biobank (application number 51671, approved August 2019) but restrictions apply to the availability of these data, which were used under license for the current study, and so are not publicly available. Data are however available from the authors upon reasonable request and with permission of the UK Biobank. UK Biobank is an open-access resource, and the study website https://www.ukbiobank.ac.uk/ has information on data availability and access procedures. Data sets used for the analysis will be made available upon reasonable request.

## Abbreviations

AD: Alzheimer’s dementia
ASP: Aspirin
BMI: Body mass index
DBP: Diastolic blood pressure
HbA1c: Hemoglobin A1c
HDL: High-density lipoprotein
LDL: Low-density lipoprotein
MetS: Metabolic syndrome
NASIDS: Non-steroidal anti-inflammatory drugs
SBP: Systolic blood pressure
SUA: Serum uric acid
TG: Triglyceride
VD: Vascular dementia
