# Supplementary material for: Investigating the nexus of metabolic syndrome, serum uric acid, and dementia risk: a prospective cohort study
Source: BMC Med. 2024 Mar 13;22:115. doi: 10.1186/s12916-024-03302-5 (PMC10938845; doi:10.1186/s12916-024-03302-5)
Supplement: Supplementary file 1 — Additional file 1: Table S1. The association between metabolic syndrome and risk of dementia after lagging for 2 years. Figure S1. Non-linear Association Between Metabolic Syndrome Components and Vascular Dementia Risk. [file 12916_2024_3302_MOESM1_ESM.doc]

**Prospective Cohort Study: Investigating the Nexus of Metabolic Syndrome, Serum Uric Acid, and Dementia Risk**

**Tara SR Chen^1,2,3^, NingNing Mi^5^, Hubert Yuenhei Lao^4^, Chenyu Wang^6^, Wai Leung Ambrose Lo^7^, YuRong Mao^1^, Yan Tang^1^, Zhong Pei*, Jin-Qiu Yuan*, Dong-Feng Huang***

1. Department of Rehabilitation Medicine, The Seventh Affiliated Hospital, Sun Yat-Sen University, Shenzhen; Guangdong Engineering and Technology Research Centre for Rehabilitation Medicine and Translation, Guangdong, China; WHO Collaborating Centre for Rehabilitation CHN-50
2. Department of Neurology, The First Affiliated Hospital, Guangdong Provincial Key Laboratory of Diagnosis and Treatment of Major Neurological Diseases; National Key Clinical Department and Key Discipline of Neurology, Sun Yat-Sen University, Guangzhou, 510080, China
3. Clinical Research Centre, The Seventh Affiliated Hospital, Sun Yat-sen University, Shenzhen, Guangdong, China; Scientific Research Center, The Seventh Affiliated Hospital, Sun Yat-Sen University, Shenzhen, Guangdong, China
4. State Key Laboratory of Ophthalmology, Zhongshan Ophthalmic Centre, Sun Yat-Sen University, Guangdong Provincial Key Laboratory of Ophthalmology and Visual Science, Guangzhou 510060, China; WHO Collaborating Centre for Eye Care and Vision CHN-151
5. The First School of Clinical Medicine, Lanzhou University, Lanzhou, Gansu, China
6. Brain and Mind Centre, The University of Sydney, Australia
7. Department of Rehabilitation Medicine, The First Affiliated Hospital, Sun Yat-Sen University, Guangzhou, China

**Correspondence**

**Professor DongFeng Huang Department of Rehabilitation Medicine, The Seventh Affiliated Hospital, Sun Yat-Sen University, Shenzhen, Guangdong, 518107, China*

*Guangdong Engineering and Technology Research Center for Rehabilitation Medicine and Translation, Guangdong, 518107,China(* [*huangdf_sysu@163.com*](mailto:huangdf_sysu@163.com)*)*

*Professor JinQiu Yuan *Clinical Research Center, The Seventh Affiliated Hospital, Sun Yat-sen University, Shenzhen, Guangdong, China; Scientific Research Center, The Seventh Affiliated Hospital, Sun Yat-sen University, Shenzhen, Guangdong,518107， China (*[*yuanjq5@mail.sysu.edu.cn*](mailto:yuanjq5@mail.sysu.edu.cn) *)*

**Professor Zhong Pei Department of Neurology, The First Affiliated Hospital, Guangdong Provincial Key Laboratory of Diagnosis and Treatment of Major Neurological Diseases; National Key Clinical Department and Key Discipline of Neurology, Sun Yat-sen University, Guangzhou, 510080, China (*[*peizhong@mail.sysu.edu.cn*](mailto:peizhong@mail.sysu.edu.cn) *)*

SUPPLEMENTARY APPENDIX

| **Table S1. The association between metabolic syndrome and risk of dementia after lagging for 2 years** | | | | | | | | |
| --- | --- | --- | --- | --- | --- | --- | --- | --- |
| **Variable** | **All-cause dementia** | |  | **Alzheimer’s disease** | |  | **Vascular dementia** | |
|  | Non-Mets | Mets |  | Non-Mets | Mets |  | Non-Mets | Mets |
| Model 1, HR (95% CI) | 1.00 (Ref) | 1.25 (1.19-1.32) * |  | 1.00 (Ref) | 1.12 (1.03-1.21) * |  | 1.00 (Ref) | 1.63 (1.47-1.82) * |
| Model 2, HR (95% CI) | 1.00 (Ref) | 1.12 (1.06-1.18) * |  | 1.00 (Ref) | 1.02 (0.94-1.11) |  | 1.00 (Ref) | 1.41 (1.26-1.57) * |
| Model 3, HR (95% CI) | 1.00 (Ref) | 1.07 (1.01,1.13) * |  | 1.00 (Ref) | 1 (0.92-1.09) |  | 1.00 (Ref) | 1.28 (1.15-1.43) * |

Model 1, stratified by age, gender and UK Biobank assessment centre.

Model 2, additionally adjusted for race (white or other), index of multiple deprivation (a measure of socioeconomic status), smoking status (never smoked, previous smoker, current smoker), alcohol consumption (never or special occasions only, one to three times a month, one to four times a week, daily or almost daily), physical activity (high, low, moderate or unknown/missing), portions of fruit and vegetable intake (<5 portions per day, ≥5 portions per day, or unknown/missing).

Model 3, fully adjusted model additionally adjusted for regular medications [multivitamin use (yes or no),mineral supplement (yes or no), non-steroidal anti-inflammatory drugs (yes or no), aspirin (yes or no)], and history of Alzheimer's disease/dementia (yes or no).


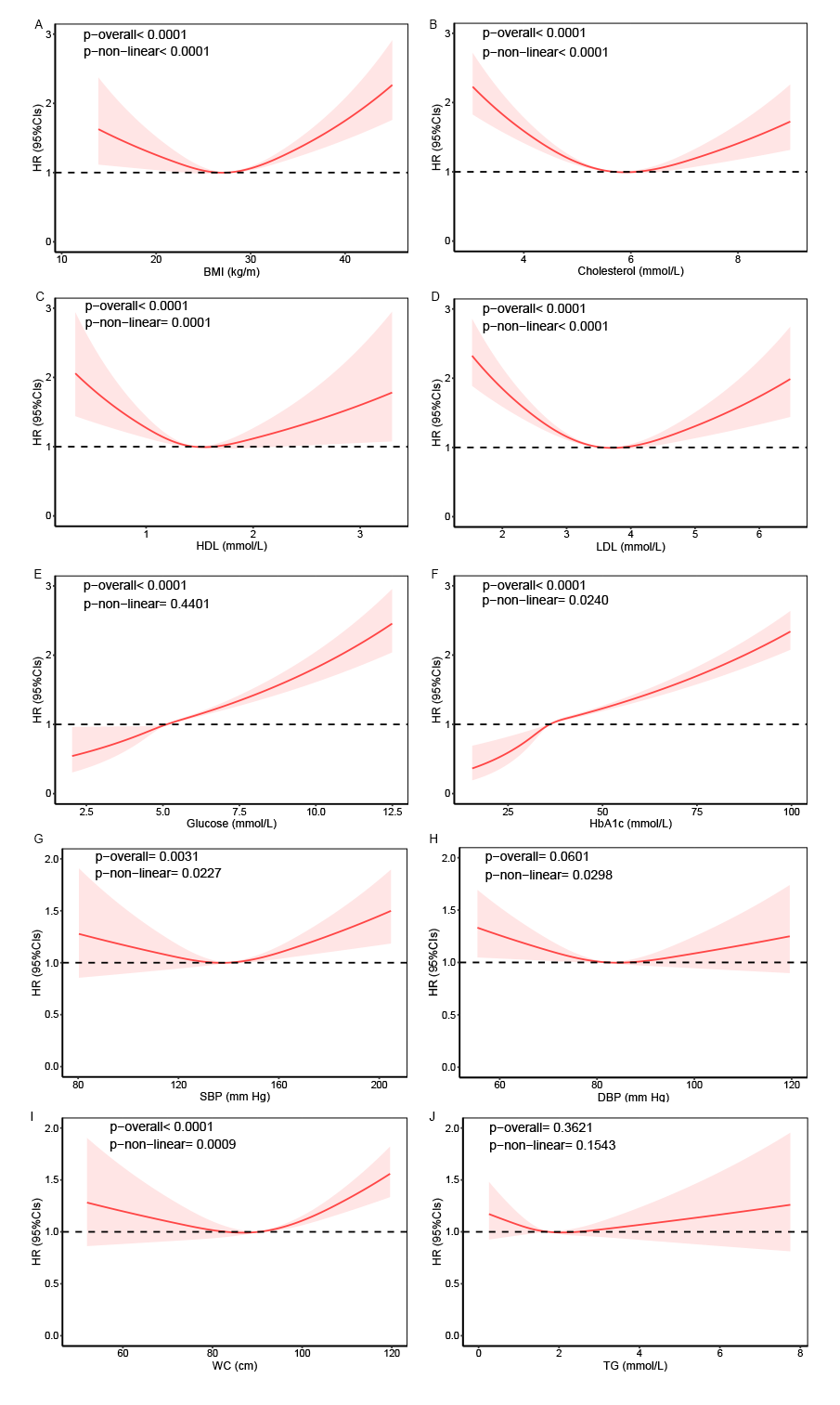


**Figure S1. Non-linear Association Between Metabolic Syndrome Components and Vascular Dementia Risk.** Restricted cubic spline models, with knots at the 10th, 50th, and 90th percentiles, were employed. Reference levels (HR fixed at 1.0) for each plot:

(A) BMI: 27.42 kg/m²; (B) Cholesterol: 5.69 mmol/L; (C) HDL: 1.45 mmol/L; (D) LDL: 3.56 mmol/L; (E) Glucose: 5.12 mmol/L; (F) HbA1c: 36.08 mmol/L; (G) SBP: 139.6 mm Hg; (H) DBP: 82.25 mm Hg; (I) WC: 90.27 cm; (J) TG: 1.74 mmol/L Adjustments were made for age, gender, and UK Biobank assessment centres. Additional adjustments included race, index of multiple deprivation, smoking status, alcohol consumption, physical activity, portions of fruit and vegetable intake, regular medications (multivitamin use, mineral supplement, non-steroidal anti-inflammatory drugs, and aspirin), and history of dementia.
